# Supplementary material for: The effect of clinically relevant beta-lactam, aminoglycoside, and quinolone antibiotics on bacterial extracellular vesicle release from E. coli
Source: bioRxiv. 2023 Nov 22:2023.11.22.568081. Preprint. [Version 1] doi: 10.1101/2023.11.22.568081 (PMC10690228; doi:10.1101/2023.11.22.568081)
Supplement: 1 [file NIHPP2023.11.22.568081v1-supplement-1.pdf]

bioRxiv preprint doi: https://doi.org/10.1101/2023.11.22.568081; this version posted November 22, 2023. The copyright holder for this preprint (which was not certified by peer review) is the author/funder, who has granted bioRxiv a license to display the preprint in perpetuity. It is made available under aCC-BY 4.0 International license.

Supplementary Data

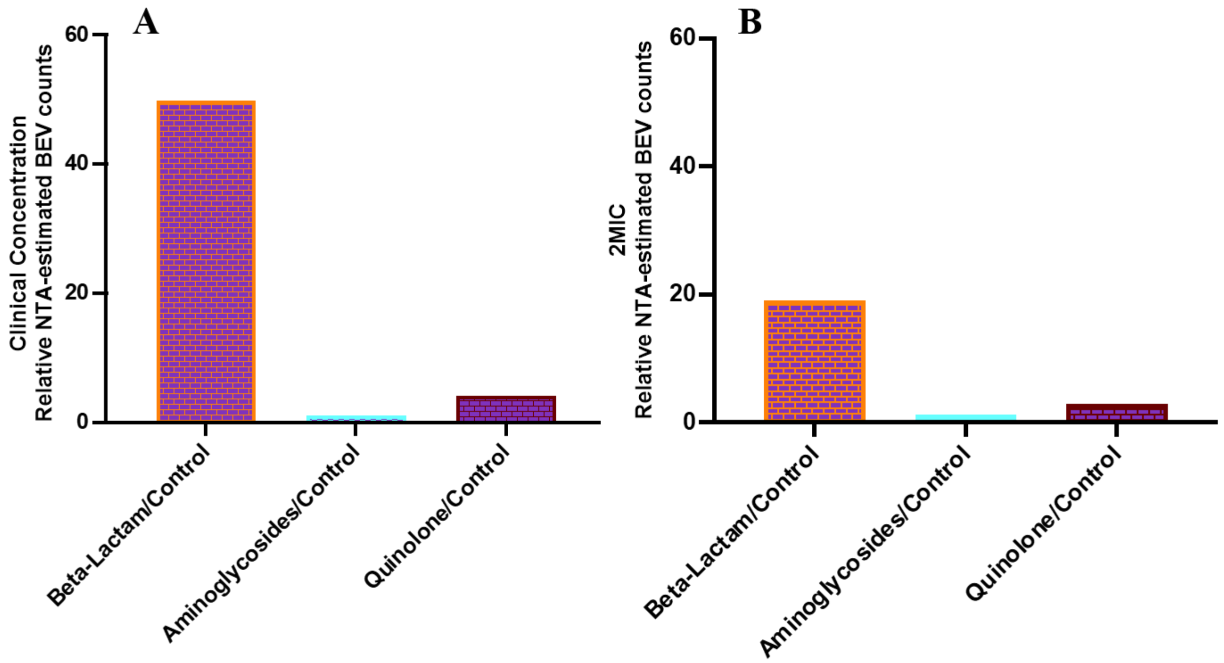

Figure S1. Relative BEV counts determined from average NTA data. Relative BEV counts were determined using the average NTA-derived particle counts for each class of antibiotic compared to control (no antibiotic) for clinical concentrations (A) and 2MIC (B).

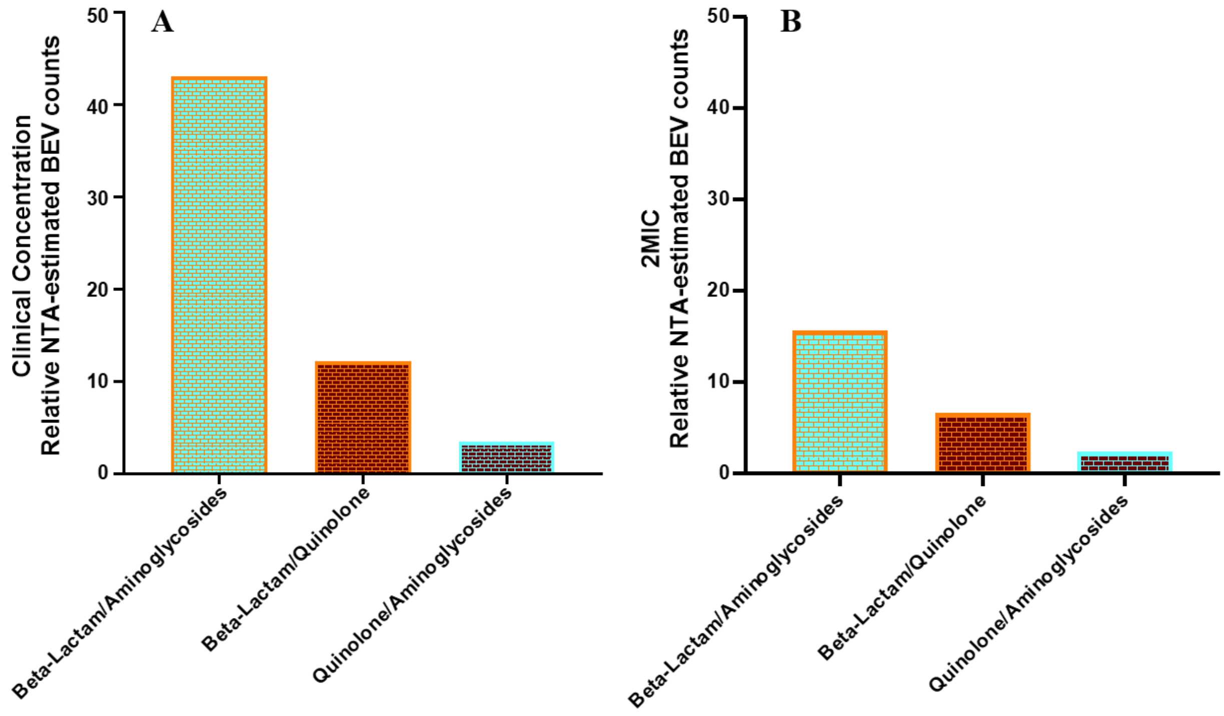

Figure S2. Relative BEV counts determined from average NTA data for beta-lactam antibiotics vs aminoglycosides, beta-lactam antibiotics vs quinolone, and quinolone vs aminoglycosides at clinical concentrations (A) and 2MIC (B).
